# RETRACTED: El-Hamshary et al. Preparation and Characterization of Nanofibrous Scaffolds of Ag/Vanadate Hydroxyapatite Encapsulated into Polycaprolactone: Morphology, Mechanical, and In Vitro Cells Adhesion. *Polymers* 2021, *13*, 1327

**DOI:** 10.3390/polym17070973

**Published:** 2025-04-03

**Authors:** Hany El-Hamshary, Mehrez E. El-Naggar, Ayman El-Faham, M. A. Abu-Saied, M. K. Ahmed, Mosaed Al-Sahly

**Affiliations:** 1 Chemistry Department, College of Science, King Saud University, P.O. Box 2455, Riyadh 11451, Saudi Arabia; aelfaham@ksu.edu.sa (A.E.-F.); 433107098@student.ksu.edu.sa (M.A.-S.); 2 Department of Chemistry, Faculty of Science, Tanta University, Tanta 31527, Egypt; 3 Textile Research Division, National Research Centre, Dokki, Cairo 12622, Egypt; 4 Department of Chemistry, Faculty of Science, Alexandria University, P.O. Box 426, Ibrahimia, Alexandria 21321, Egypt; 5 Polymeric Materials Research Department, Advanced Technology and New Materials Research Institute, City of Scientific Research and Technological Applications (SRTA-CITY), New Borg El-Arab City 21934, Alexandria, Egypt; mouhamedabdelrehem@yahoo.com; 6 Department of Physics, Faculty of Science, Suez University, Suez 43518, Egypt; mkaa-eg@hotmail.com; 7 Faculty of Nanotechnology for Postgraduate Studies, Cairo University, El-Sheikh Zayed 12588, Egypt

The journal retracts the article titled “Preparation and Characterization of Nanofibrous Scaffolds of Ag/Vanadate Hydroxyapatite Encapsulated into Polycaprolactone: Morphology, Mechanical, and In Vitro Cells Adhesion” [1], cited above.

Following publication, concerns have been brought to the attention of the Editorial Office relating to image irregularities contained within this article [1].

Adhering to our standard procedure, an investigation was conducted by the Editorial Office and the Editorial Board, which confirmed a high level of similarity between a figure presented in this article [1] and earlier publications by the same authors [2,3], showing different experimental conditions. Following a review of the material provided by the authors, the Editorial Board has lost confidence in the integrity of the findings and has decided to retract this publication [1], as per MDPI’s retraction policy (https://www.mdpi.com/ethics#_bookmark30; accessed on 1 December 2024).

This retraction was approved by the Editor-in-Chief of *Polymers*.

The authors did not agree with this retraction.
